# Pilot development of diagnostic tools for lower limb apophyseal injuries in children and adolescents

**DOI:** 10.7717/peerj.18101

**Published:** 2024-09-20

**Authors:** Joseph Brooks, Antoni Caserta, Kade Paterson, Kelly-Ann Bowles, Terry Haines, Cylie Williams

**Affiliations:** 1 School of Primary and Allied Health Care, Monash University, Frankston, VIC, Australia; 2 Centre for Health, Exercise and Sports Medicine, Faculty of Medicine Dentistry & Health Sciences, University of Melbourne, Melbourne, Victoria, Australia

**Keywords:** Delphi technique, Foot, Knee, Telehealth, Children, Apophysitis

## Abstract

**Introduction:**

Lower limb apophyseal injuries commonly occur in children and adolescents with unknown incidence and prevalence. These conditions are considered benign, but impact children and adolescents quality of life and can lead to sport withdrawal at a crucial time. The primary aim of this research was to develop self-administered tools for two of the most common apophyseal injuries. The secondary aim was to test the sensitivity and specificity of the tools.

**Methods:**

Study 1 used a three round online Delphi panel (*n* = 8), with expert consensus supported by robust literature. This panel developed a self-administered screening tool for calcaneal (Sever’s disease) and tibial tuberosity (Osgood-Schlatter’s disease) apophysitis. Study 2 tested the sensitivity and specificity of these developed tools with parents and children (*n* = 63) with concurrent clinical examination by a health professional. An initial sample size for Study 2 was set at 155 children however this was impacted by COVID-19 and recruitment was halted.

**Results:**

Both tools had excellent diagnostic accuracy with an area under the curve of 83% (95% confidence interval = 0.70 to 0.95) for the posterior heel (calcaneal apophysitis) tool and 93% (95% confidence interval = 0.80 to 1.00) for the anterior of knee (tibial tuberosity apophysitis) tool using the pilot data from the 63 children.

**Conclusions:**

These tools may also enhance opportunities for clinicians and health service providers with pre-clinical screening to reduce wait list time and encourage low cost, self-administered management where indicated. These findings may enable large epidemiological studies to identify populations and calculate incidence and prevalence of these conditions using self report.

## Introduction

Screening tools are becoming more popular in incidence studies (Narzisi et al., 2020), telehealth treatment sessions (Jones et al., 2009) or to assist health professionals in specialist services care escalation (Williams, Tinley & Curtin, 2010). When developed appropriately, and with good sensitivity and specificity, tools reduce participant and health professional burden. In particular, self-administered tools are particularly advantageous, due to being relatively inexpensive to administer. Self-administered tools have been used in paediatric populations for screening or epidemiological studies in areas such as depression (Richardson et al., 2010), sleep problems (Spruyt & Gozal, 2011) or quality of life relating to particular health conditions (DeWalt et al., 2015). No such tools are available for some of the most common conditions in childhood (de Lucena, dos Santos Gomes & Guerra, 2011; Kujala, Kvist & Heinonen, 1985), such as for children with apophyseal injuries.

These injuries are often referred to by their eponymous names, with the two most common calcaneal apophysitis (Sever’s Disease) and tibial tuberosity apophysitis (Osgood Schlatters disease) (De Inocencio, 2004). Pain location is generally at the secondary apophyseal site and corresponding tendon attachment. These common conditions have varied aetiology, and may occur any times between 8–15 years (Kujala, Kvist & Heinonen, 1985; Ogden et al., 2004). It was previously thought that conditions were self-limiting, and fully resolve once skeletal maturity was reached. Growing evidence suggests this may not be the case in all type of apophyseal injuries (Guldhammer et al., 2019). These injuries are reported by children to have considerable impacts on their lives (Guldhammer et al., 2019; James, Williams & Haines, 2016).

Apophyseal injuries are aggravated by particular sports involving frequent jumping or running (Itoh et al., 2018; Madden & Mellion, 1996). However, even children not participating in these sports describe these conditions at times (James et al., 2015). This poses challenges in truly understanding the conditions and studying their frequency. If there is limited awareness of frequency or impact of common childhood conditions, the development of these may impact on a child’s abilities to meet physical activity recommendations. This becomes particularly relevant with the rise of childhood obesity (Li et al., 2019).

The primary aim of this research was to develop self-administered tools for the two most common apophyseal injuries. Secondary aims were to test the tools sensitivity and specificity. It was our hypothesis that conducting this research using a staged approach of expert clinicians in diagnosis, combined with testing this tool in clinical practice would result in a tool with high sensitivity and specificity.

## Method

This research comprised two studies with different methodologies. Study 1 used the Delphi technique (Vernon, 2009) which was modified to run online with experts, in order to develop a self-administered screening tool for calcaneal and tibial tuberosity apophysitis. This methodology involve the recruitment of experts with sequential anonymised survey rounds to develop consensus or agreement (Jones & Hunter, 1995). This method is particular of use in the absence of uniform guidelines or tools (Vernon, 2009), with small to large groups where anonymity is enable through online methods (Sinha, Smyth & Williamson, 2011). We have adhered where possible to the guidelines specific to the study designs (Bossuyt et al., 2015; Jünger et al., 2017) and outlined deviations to this within the limitations.

Study 2 tested sensitivity and specificity with clinical examination and concurrent self-completion of the tool with qualified health professionals and families. No clinicians (medical doctors, physiotherapists or podiatrists) participated in both Study 1 (as participants in the Delphi) and Study 2 (as recruiters and assessors of children). Research was approved by Monash University Human Research Ethics committee (14294 & 20156).

### Participants and procedures for Study 1

Study 1 participants were Australian health professionals (medical doctors, physiotherapists and podiatrists) who had expertise in diagnosis, treatment or research of lower limb apophyseal injuries. All participants had similar access to appropriate diagnostic methods or ability to escalate care where appropriate. Expertise was assumed through additional training (such as medical fellowships or allied health credential received from peak bodies) or leading research specific to apophyseal injuries published within past 5 years.

Potential participants were invited *via* email. Following written and informed consent, they were emailed the online Round 1 of Delphi survey.

**Figure 1:**
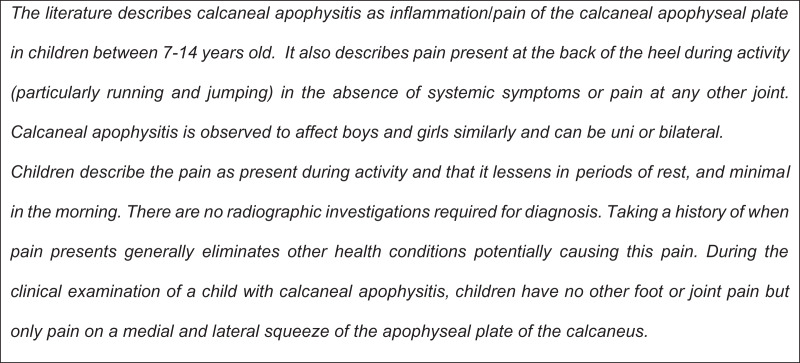
Text description of calcaneal apophysitis.

**Figure 2:**
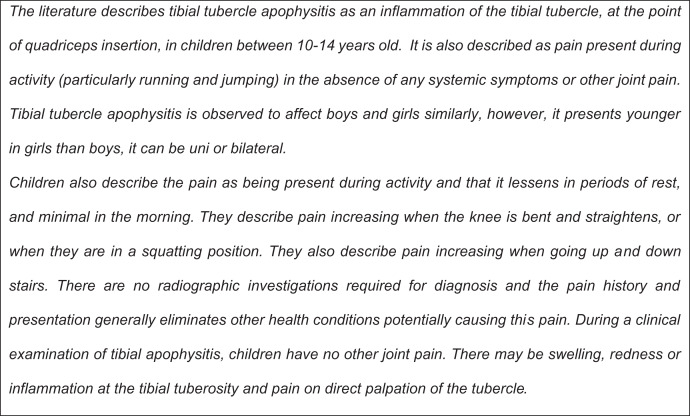
Text description of tibial tubercle apophysitis.

Round 1 collected participant demographics including perceived conflicts of interest or bias towards self-administered diagnostic tools, gender, years of practice and profession. This round also asked the participants to read a description of both calcaneal apophysitis and tibial tuberosity apophysitis developed by the research team (JB, AC, TH, CW), based on recent systematic reviews (James, Williams & Haines, 2013; Ladenhauf, Seitlinger & Green, 2020). These two descriptions are in Figs. 1 and 2. Participants were asked two similarly worded open ended questions about each condition:

1. After reading the above description of the above condition, please list all of the questions you ask the child and parents when a child presents with heel/knee pain to aid your diagnosis of apophysitis?

2. After reading the above description of clinical examination of apophysitis to aid diagnosis only, what examinations do you routinely undertake to confirm the diagnosis?

Participants were encouraged that they were able to repeat or add additional information from the developed definitions.

Participants completed their responses within 4 weeks. Their responses were independently summarised by two members of the research team (JB and CW) and where discord, a third member assisted (AC).

As the purpose was to develop a self-administered tool for diagnosis, the responses were re-presented to participants in question form. Where participant responses of questions or assessments in Round 1 were similar as 70% or greater, these were accepted as consensus. These responses were provided to participants in Round 2, and participants were able to provide feedback on the question wording only, not if it should remain in any resultant tool.

Questions or assessments described by 50–69% of participants were refined and represented to participants in Round 2 for agreement rating. Questions or assessments described by less 50% of participants did not progress to the Round 2.

In Round 2, participants were asked to indicate agreement on the generated questions through a five-point Likert scale (Strongly Agree, Agree, Neither Agree or Disagree, Disagree or Strongly Disagree). They were also invited to provide comment about question phrasing. Similar to Round 1, any questions with 70% or greater agreement were included within the tool. Questions with 50–69% agreement were re-phrased according to feedback and taken back to participants in Round 3. Any outstanding questions not meeting agreement at the end of Round 3 were removed from the tool. The final tool was used in Study 2 without further modification.

### Participants and procedures for Study 2

The Study 2 participants were physiotherapists and podiatrists known through clinical and institutional networks as working with children, and working in private practices in Victoria, Tasmania and Queensland, were invited to participate in the tool validation component. A whole of practice invitation was made and rather than individual clinicians. We used a consecutive sampling technique where participating clinicians and their practice staff were provided with instructions to sequentially invite all children who presented to their clinic for front of knee pain or back of heel pain. There were no limiters to age, only location of pain. Both physiotherapists and podiatrists gave written informed consent, parent’s implied informed consent through informed completion of the survey, and children assented.

Each clinician participating provided their qualifications, years of practice and an estimate of the number of children seen monthly with the conditions of interest. The finalised tool from the Delphi panel was presented as a one-page handout with either six (heel pain) or seven (knee pain) questions. To complete this tool, the child or their parent ticked a “yes” or “no” for each question (Table 1)

**Table 1:** Heel and knee tools.

If your child has back of the heel pain	Yes	No
Has your child had heel pain for less than 3 months?	□ (1)	□ (0)
Does your child have pain at any other joints *i.e*., hips, knees, hands?*	□ (0)	□ (1)
Is your child between the ages of 8–14?	□ (1)	□ (0)
Does the pain resolve later that evening, or the following day when not being physically active?	□ (1)	□ (0)
Is there pain in the area highlighted in the picture 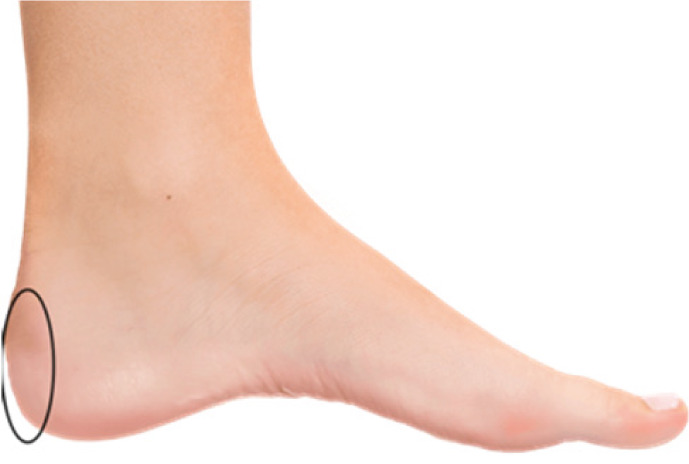	□ (1)	□ (0)
If you look at the picture and squeeze the back of the heel in a similar way, is it painful? 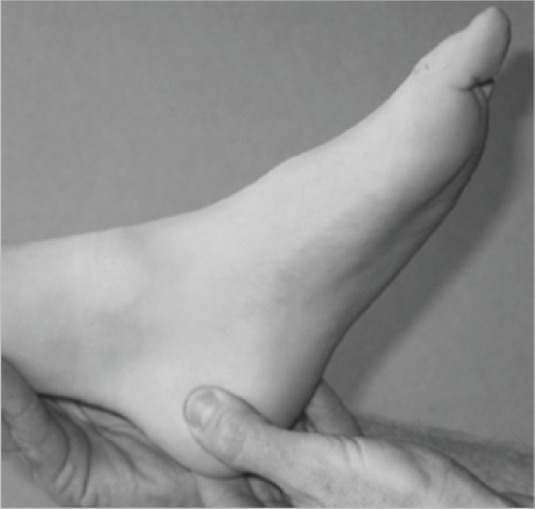	□ (1)	□ (0)

**Note:**

* Reverse scored.

All participating clinicians were given an information pack that included self-sealing opaque envelopes, information/consent forms, and a tool with questions about back of heel pain (calcaneal apophysitis) on one side of the article and front of knee pain (tibial tuberosity apophysitis) on the other. When parents identified their child was at the clinic for heel or front of knee pain, reception staff gave them the information/consent form while they were in the waiting room. If they consented, the parents and children filled out the relevant side of the tool, sealed it in an opaque envelope, and handed this envelope to the clinician. After the consultation, the clinician recorded the diagnosis, along with the child’s gender and year of birth, on the front of the sealed envelope. If additional diagnostic tests were needed to assist the clinician, the envelope was stored until a diagnosis was confirmed. Once the diagnosis was confirmed, the clinician completed the front of the envelope, and placed it inside a larger opaque envelope with other sealed responses from other participants and sent all back to the research team. This process ensured that the participating clinicians remained unaware of the child and parent responses.

### Data analysis

Once the research team received the responses, they were separated into heel or knee diagnoses for tool score calculation and data analysis. Each diagnosis was categorized as (1) an apophyseal injury, or (2) not an apophyseal injury or an apophyseal injury complicated by a co-diagnosis (*e.g.* calcaneal apophysitis and suspected subtalar joint coalition). Each tool scores were calculated out of 6 (back of heel tool) or 7 (anterior of knee tool), including the negatively scored responses (*i.e*. Does your child have any pain at any other joints). Scores between apophysitis diagnosis and other diagnoses were initially described by the median, interquartile range (IQR) and range. Data was entered into Microsoft® Excel for Mac (Version 16.87), then transferred to Stata (Release 15) for calculation of sensitivity, specificity and generation of figures. We calculated sensitivity, where a true positive was identified by the tool (confirmed by assessment), and specificity, probability where a true negative diagnosis (confirmed by assessment) was correctly identified by the tool. We also calculated positive predictive values where the tool identified apophyseal injuries and the negative predictive values, where the probability the tool predicted the injury was not related to an apophyseal injury or an injury complicated by another. We plotted a receiver operating characteristic (ROC) curve analysis for both heel and knee responses to determine optimal cut points for tools (*i.e*., the total score with the highest sensitivity and specificity). The ROC curve plots the sensitivity (y axis) against 1 minus the specificity (x-axis) responses resulting from the negative question responses. The area under the curve (AUC) provides a summary measure of diagnostic accuracy across the spectrum of responses, where 0.5 is the level of accuracy that is afforded by random chance and was considered by investigators to have no utility for classification of an apophyseal injury, 0.7–0.8 is acceptable, 0.8–0.9 is excellent, over 0.9 considered outstanding (Hosmer & Lemeshow, 2000). The Youden index (*J*) was calculated (sensitivity + specificity − 1) at each score as an index of performance of the tools for the population with and without the condition at each score. This was calculated to support optimal score choice for diagnostic accuracy and minimised the number of false positives, or to potential of missing a true diagnosis (Youden, 1950).

A preplanned sample size of 155 children was determined based on a 20% prevalence of apophyseal injuries presenting in all children’s musculoskeletal injuries in a general clinics (de Inocencio, 1998) for these conditions, with a power of 0.81 to detect a sensitivity and specificity of >90% for clinical condition of interest (Bujang & Adnan, 2016). Due to the current coronavirus pandemic, data collection ceased early due to limitations on face to face services at each location and unknown return to normal practice timeframes.

## Results

There were 13 expert panellists invited, medical specialists (*n* = 4), physiotherapists (*n* = 5) and podiatrists (*n* = 4). From these, eight participants consented and completed two rounds, and seven completed the final round. Participants were two doctors (sports medicine or paediatric orthopaedic specialisation), three physiotherapists, and three podiatrists. All participants had treated children with apophyseal injuries for greater than 10 years, there were five males (63% of 8), six had completed their PhD, or a professional doctorate, and medical specialists held more than one professional medical fellowship (*e.g*., Fellow of Australian Orthopaedic Association or Fellow of the Australasian College for Emergency Medicine).

The final tools were broken into distinct question categories relating to establishing the history of acute pain, age group of interest, history of injury or it’s resolution and distinct pain locations in the absence of other joint pain. The final two tools are presented (Table 1).

Six clinics, with nine podiatrists and two physiotherapists collected data over a 6-month period from October 2019 until March 2020. All clinicians had a minimum of 7 years’ experience and assessed and treated a minimum of four children per month in the target age group. These clinicians enabled 63 (47 heel and 16 knee) parent/child tool completions and subsequent assessments.

Out of the 47 children with posterior of heel pain, 28 (59%) received a diagnosis of calcaneal apophysitis by their treating therapist, five (11%) were diagnosed with calcaneal apophysitis combined with an additional foot condition, and 14 (30%) children had other diagnoses including tendinopathy, retrocalcaneal bursitis or tarsal coalition. Children who had a diagnosis of calcaneal apophysitis had a median (IQR) score of 5 (5, 6). Children with another condition causing their heel pain or calcaneal apophysitis in combination with another condition had a median (IQR) score of 4 (3, 5). Out of the 16 children with knee pain, 12 (75%) had tibial tuberosity apophysitis, and four (25%) had other knee conditions including a high index of suspicion of juvenile idiopathic arthritis or chondromalacia patella. Children with tibial tuberosity apophysitis had a median (IQR) score of 5 (5, 6). Children with other knee pain had a median (IQR) score of 4 (3,5).

**Figure 3:**
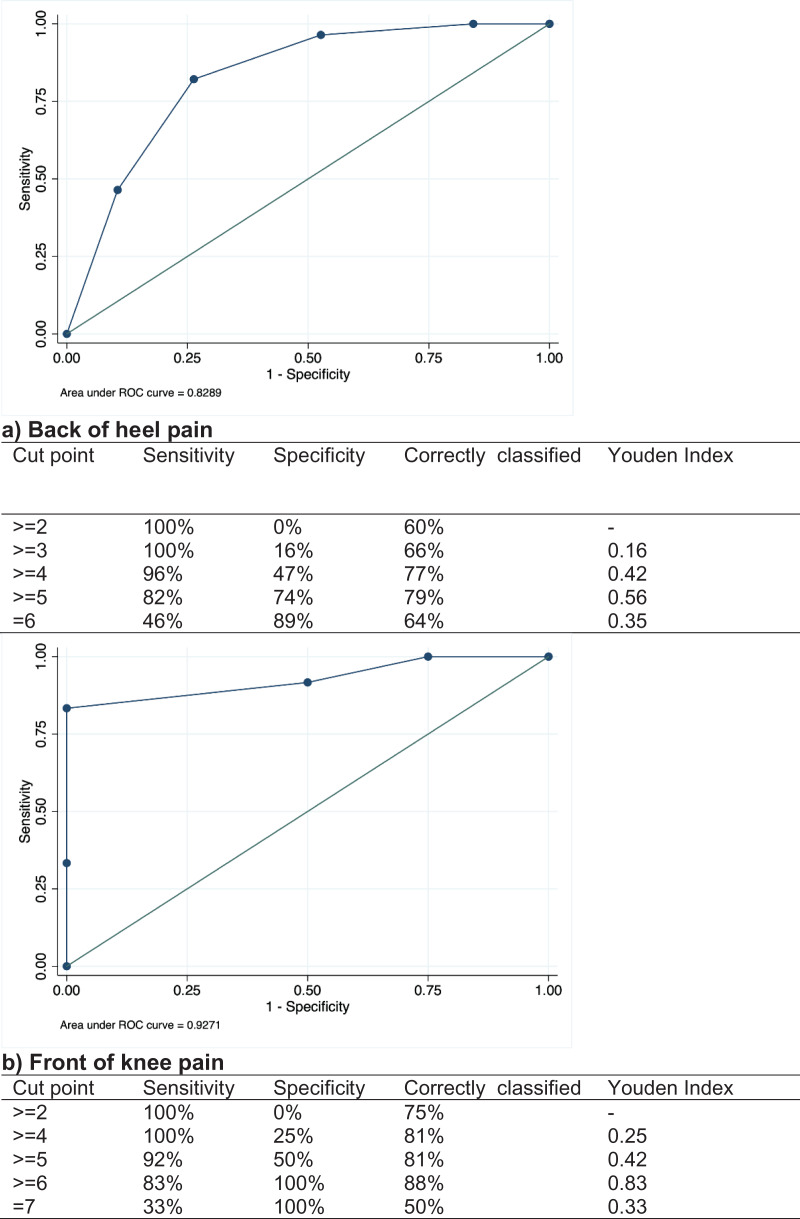
ROC curves: (A) back of heel pain and (B) front of knee pain.

The ROC curves and sensitivity and specificity relating to scores are presented in Figs. 3A and 3B. For the heel pain, the tool correctly identified calcaneal apophysitis 78.7% (*J* = 0.56) of the time where the score was five or higher. For knee pain, the tool correctly identified tibial tuberosity apophysitis correctly in 87.5% (*J* = 0.83) of cases where the score was 6 or greater. Both tools had excellent diagnostic accuracy with an AUC of 83% (95% confidence interval = 0.70 to 0.95) for the heel tool, and 93% (95% confidence interval = 0.80 to 1.00) for the knee tool. Therefore, the cut point of five for the posterior heel tool and six for the anterior knee tool maximises the Youden Index.

## Discussion

This study outlines the promising results of a self-administered diagnostic questionnaire tool for the two most common lower limb apophyseal injuries. Based on the results, these tools in their current form were developed through rigorous expert consensus and trialled with children, their parents and clinicians. The tools demonstrate acceptable validity and should be considered for further large-scale testing.

These tools, and the results of testing, present many research and clinical opportunities such as the potential for use to assist in medical waitlist triage, to alert parents when a timely appointment should be sought, and where first line, low cost management and education can be self-implemented. Cut points of each tool, their sensitivity and specificity show promise as a cost effective and simple mechanism to identify where a child with foot or knee pain may be able to initial simple home-based treatments for an apophyseal injury. Where a child scores 5 or greater on the heel tool, or 6 or greater on the knee tool, they may be provided with simple home-based strategies, such as ice or short-term load management. Inversely, a low score on the tool should indicate a potentially more serious reason for pain, or prompt a parent to seek treatment for their child from a clinician. Questions were particularly elicited from clinicians to ensure conditions such as juvenile idiopathic arthritis were unlikely to be missed by this tool. At present, these tools are the first of their kind for these sorts of injuries in children. Similar such self-administered tools have been developed for adults and children, used successfully for conditions that discrete symptoms such as with asthma (Jenkins et al., 1996) but poorly for conditions where there are wide variations in presentation without clear indicators, such as periodontal disease (Vered & Sgan-Cohen, 2003).

There are a lack of known self-administered tools to enable comparison in identification or diagnosis of sporting injuries. This creates unique opportunities to further examine the validity with larger populations. Understanding how the tools perform with larger populations will provide the confidence to embed them within widescale epidemiological studies. This may allow the tool to be used online, or over the phone, to understand just how many young people have these conditions and if there is any the impact of these conditions on their physical activity. Embedding these tools in health services or in population-based studies may also allow a standardised approach to prevalence and incidence data collection in different health settings. The unknown incidence and prevalence of these conditions, challenge decision makers and trialists on where to direct research funds for interventions relating to childhood injury and obesity. The numbers of children presenting to small health care department indicate a need for service delivery, but the true impact is unknown, with numbers presenting to diverse health care providers (Kujala, Kvist & Heinonen, 1985; Lau, Mahadev & Hui, 2008; Wiegerinck et al., 2014).

Tool and clinical pathways are continually being refined to minimise overtreatment, ensure the right referral to the right specialist, at the right time, and only when needed (Jessup & Buchbinder, 2018; Von Kobyletzki et al., 2012). Given musculoskeletal pain in children and adolescents make up a significant number of general practitioner presentations, the use of these tools may have multiple benefits (Henschke et al., 2014). Future research may focus on their use as a mechanism to understand if care should be managed in specialist clinics and if so, which clinic or specialisation should be considered. This is particularly important where a child may have an underlying inflammatory conditions, that each of the tools may identify through the screening questions indication multi-joint pain over a greater than 3 month period (Chen, Thrower & Garrood, 2020). With the increasing use of online health advice and shift to increase in telehealth, the lower limb apophyseal injury tools have the potential to guide both parents and clinicians to an accurate diagnosis or when tailored health advice is required.

This study’s strength was its mixed methodology. The Delphi technique, although deemed lower in the evidence pyramid, remains a valid method where there is limited theory or empirical literature within the relevant context or setting, despite the small sample size (Adler & Ziglio, 1995). We supplemented the small sample size with presentation of robust theory which we believe mitigated concerns with panel size. Our research and the tool development were also strengthened through the multi-disciplinary partnership with parents, children, and clinicians blinded to the parent/child responses. This enabled data collection outside of any influential relationship with the research team. The primary limitation was the lack of statistical power resulting from a lower than anticipated sample size of children, increasing any confidence intervals that would have been constructed around our results. The continued challenge of the coronavirus pandemic, and face to face data collection in Australia, meant that it was unfeasible to continue or resume the study to date. However, these promising results indicate that future larger scale use of this tool should be considered both clinically and in research to determine its validity with larger and appropriately powered sample sizes where knee and heel data can be individually collected. Data validation sets also may be one way to test this in the future, however they have a potential to overestimate accuracy (Haines et al., 2007). The methodology used within the study design also demonstrated methodological feasibility with engaged and experienced clinicians. This may be expanded in the future when clinical settings return to consistent face to face care.

## Conclusion

This study outlines the development of two promising diagnostic tools for the two most common lower limb apophyseal injuries; calcaneal apophysitis and tibial tuberosity apophysitis. These may enable large epidemiological studies to estimate the prevalence and incidence of these conditions. Similarly, these tools may enhance opportunities for pre-clinical screening to reduce waitlist times and encourage low cost, self-administered management where indicated.

## Supplemental Information

10.7717/peerj.18101/supp-1 Supplemental Information 1 Anonymised raw data.

10.7717/peerj.18101/supp-2 Supplemental Information 2 Statements generated by experts through the three rounds with green shading representing acceptance, orange shading indicating moving to the next round and red shading indicating statement was discarded.

## Additional Information and Declarations

### Field Study Permissions

The following information was supplied relating to field study approvals (*i.e*., approving body and any reference numbers): Individual private practice collected data. All provided consent as part of the consent process and ethical application through Monash University. This was part of the ethics application

### Data Availability

The following information was supplied regarding data availability: The anonymised raw data from sensitivity and specificity is available in the Supplemental File.
