# Supplementary material for: Pilot development of diagnostic tools for lower limb apophyseal injuries in children and adolescents
Source: PeerJ. 2024 Sep 20;12:e18101. doi: 10.7717/peerj.18101 (PMC11418814; doi:10.7717/peerj.18101)
Supplement: Supplemental Information 2 [file peerj-12-18101-s002.docx]

Appendix 1. Statements generated by experts through the three rounds with green shading representing acceptance, orange shading indicating moving to the next round and red shading indicating statement was discarded.

| Statements | Round 1  n=8 | Round 2  n=8 | Round 3  n=7 |
| --- | --- | --- | --- |
| **Question 1: Questions asked to guide a remote diagnosis of calcaneal apophysitis** | | |  |
| Age range consistent with diagnosis | 63% | 63%  Age range 8-12 years | 100%  Age range 8-14 years |
| Duration of pain being short term (<3 months) | 75% |  |  |
| Posterior heel pain location consistent with apophysis | 88% |  |  |
| Pain consistent with diagnosis (i.e. not first step pain, no overnight pain) | 38% |  |  |
| Absence of any other joint pain, diagnosis or systemic symptoms of inflammatory disease | 75% |  |  |
| Swelling in area | 25% |  |  |
| Pain amount measured quantitatively | 38% |  |  |
| Pain pre and post activity | 13% |  |  |
| Pain at or near activity and resolving with rest | 63% | 63% | 86% |
| Pain occurring after a recent growth spurt | 25% |  |  |
| Pain being dull | 13% |  |  |
| History of previous heel pain | 13% |  |  |
| Meeting all developmental milestones | 25% |  |  |
| What medications the patient was taking | 25% |  |  |
| Any allergies | 25% |  |  |
| If there was a family history of heel pain | 38% |  |  |
| What sports they had played in the past | 13% |  |  |
| Footwear style preference | 38% |  |  |
| **Question 2. Assessments used to support a remote diagnosis of calcaneal apophysitis** | | |  |
| Foot posture | 13% |  |  |
| Pain on medial and lateral squeeze of the heel | 50% | 63% | 86% |
| Vibration perception/change in sensation with tuning fork | 13% |  |  |
| Thompson test (calf squeeze to test Achilles tendon integrity) | 13% |  |  |
| Absence of injury/trauma appearance | 38% |  |  |
| Presence of toe walking | 25% |  |  |
| Foot range of motion | 13% |  |  |
| Tanner scale/pre/post menarche | 13% |  |  |
| Subtalar joint range of motion | 13% |  |  |
| Silfverskiöld test for isolated gastrocnemius tightness | 13% |  |  |
| Site observation for skin changes | 13% |  |  |
| Provocation of pain during range of movement | 38% |  |  |
| Calf raise quality and endurance | 13% |  |  |
| Ability to squat | 13% |  |  |
| Appearance on ultrasound | 13% |  |  |
| **Question 3: Questions asked to guide a remote diagnosis of tibial tuberosity apophysitis** | | |  |
| Age range consistent with diagnosis | 50% | 50%  Age range 8-14 years | 100%  Age range 8-16 years |
| Pain location consistent with the diagnosis (tibial tuberosity) | 75% |  |  |
| Absence of any other joint pain, diagnosis or systemic symptoms of inflammatory disease | 75% |  |  |
| Swelling in area | 25% |  |  |
| Pain consistent with diagnosis and load | 88% |  |  |
| Uni or bilateral pain | 25% |  |  |
| How effective common treatments have been such as pain medications | 38% |  |  |
| Knee pain increase with bending tasks such as exercise, stairs or squatting | 75% |  |  |
| Knee locking or popping | 13% |  |  |
| Recent growth spurt | 25% |  |  |
| Pain amount measured quantitatively | 13% |  |  |
| Sport types | 25% |  |  |
| Pain not related with trauma, hit or kick to the knee | 50% | 75% |  |
| **Question 4. Assessments used to support a remote diagnosis of tibial tuberosity apophysitis** | | |  |
| Hip and knee joint range of motion | 25% |  |  |
| Pain on direct palpation of the tibial tuberosity | 63% | 75%  But with suggested language and pictorial changes | 86% |
| Ankle range of motion | 13% |  |  |
| Foot posture index-6 | 13% |  |  |
| Sit to stand | 13% |  |  |
| Ability to hop | 37% |  |  |
| Ability to squat/single leg squat | 13% |  |  |
| Apprehension and grind test | 13% |  |  |
| Pain on passive knee movement | 25% |  |  |
| Quadriceps/hamstring muscle length and strength | 25% |  |  |
| Triceps surae strength | 13% |  |  |
| Lower limb alignment | 13% |  |  |
| Ultrasound | 13% |  |  |
| Provocation loading tests | 25% |  |  |
| Double jump | 13% |  |  |
